# The emerging relationship between mucosal-associated invariant T cell populations and the onset and progression of type 1 diabetes

**DOI:** 10.3389/fimmu.2025.1602934

**Published:** 2025-06-18

**Authors:** Devin R. Fraser, Andrew W. Stadnyk

**Affiliations:** ^1^ Department of Microbiology and Immunology, Faculty of Medicine, Dalhousie University, Halifax, NS, Canada; ^2^ Department of Pediatrics, Faculty of Medicine, Dalhousie University, Halifax, NS, Canada; ^3^ Division of Gastroenterology and Nutrition, IWK Health, Halifax, NS, Canada

**Keywords:** MAIT cell, type 1 diabetes, microbiome, inflammation, interleukin

## Abstract

Type 1 diabetes (T1D) is a chronic autoimmune disorder characterized by autoreactive CD8^+^ T cells that destroy insulin-producing pancreatic β-cells. CD8^+^ T cells are unlikely to be the only cells involved in diabetes. Relatively recently described and still enigmatic, Mucosal-associated invariant T (MAIT) cells, innate-like T cells that recognize microbial-derived peptides, exist in the blood and tissues and are implicated in early immune responses. Immunological differences, some of which implicate MAIT cells, exist between individuals at different stages of T1D progression. This review explores the emerging relationship between gastrointestinal and pancreatic MAIT cell populations and the onset and progression of T1D. Early microbial colonization is critical for immune maturation, homeostasis, and MAIT cell development, and disruptions such as Caesarean delivery or antibiotic-induced dysbiosis correlate with increased T1D incidence. Diabetes progression in the diabetes-prone NOD mice is associated with reduced gut mucosal integrity, impairing the protective IL-17 and IL-22 responses of gut-resident MAIT cells and exacerbating systemic inflammation. MAIT cells recruited to the inflamed pancreas during T1D onset likely contribute to β-cell destruction through IFN-γ and granzyme B production. This hypothesis is supported by altered MAIT cell frequencies and phenotypes in individuals with T1D; MAIT cells are reduced in the blood of children recently diagnosed with T1D, potentially corresponding to pancreatic migration, while adults with long-term T1D have persistent, circulating MAIT cells with exhaustion markers. MAIT cells appear to have dual protective and pathogenic roles impacted by microbiome interactions. Understanding these relationships may inform non-invasive biomarkers for the disease.

## The emerging relationship between mucosal-associated invariant T cell populations and the onset and progression of type 1 diabetes

Mucosal-associated invariant T (MAIT) cells are a subset of innate-like T cells that account for 1-8% of T cells in human blood and mucosal tissues and 20-45% of T cells in the liver (1). Human MAIT cells are characterized by a semi-invariant αβ T cell receptor (TCR), which includes an invariant Vα7.2 chain that recognizes derivatives of the riboflavin synthesis pathway, a mechanism unique to bacteria, mycobacteria, and yeast. These derivatives trigger a MAIT cell response when presented on the monomorphic major histocompatibility complex class 1-related molecule 1 (MR1) (2).

The TCR-dependent activation of MAIT cells requires co-stimulation through CD28 and cytokines interleukin-12 (IL-12) and IL-18 (2). However, MAIT cells can be activated, albeit less robustly, independent of the TCR by direct stimulation with IL-12 and IL-18 (3). Upon activation, MAIT cells release an arsenal of proinflammatory cytokines and cytotoxic effectors, including interferon γ (IFN-γ), tumor necrosis factor (TNF), IL-17, granzyme B, and perforin. This suggests a critical role for MAIT cells in early host defense against bacterial and viral infections (4).

MAIT cells can be either circulatory or tissue-resident and exhibit distinct phenotypes and functions based on localization and activation. Most MAIT cells in human blood are CD4^-^CD8^+^, and activated MAIT cells typically adopt a type 1 or type 17 immune response, denoted MAIT1 and MAIT17, respectively. MAIT1 subsets express the T-box transcription factor TBX21 (T-bet), Eomesodermin, and B lymphocyte-induced maturation protein 1 (BLIMP-1), and secrete IFN-γ, TNF, and cytotoxic effectors granzyme B, perforin, and granulysin. MAIT17 subsets express the retinoic acid receptor-related orphan receptor gamma (RORγt), facilitating the secretion of IL-17. These subsets are not fixed; additional phenotypes and phenotype switching have been observed (5).

Tissue-resident MAIT cells have exhibited distinct functions from their circulatory counterparts. For instance, activated liver-resident MAIT cells are more polyfunctional than circulatory MAIT cells, displaying a unique tissue-repair signature that includes enhanced IL-12 signaling and increased fatty acid metabolism (3). Mucosal-resident MAIT cells exhibit increased expression of activating and proinflammatory genes, including TNF and IL-23 receptor (IL23R), compared to circulatory MAIT cells, suggesting that tissue-resident MAIT cells may be better positioned to respond to inflammation (1). The unique MAIT cell phenotypes and functions will be explored in the context of steady-state and inflammatory conditions. Against this backdrop of early descriptions of MAIT cell phenotypes, we will comment on the emerging connections being made between gut and pancreatic MAIT cell populations and the onset and progression of type 1 diabetes.

Type 1 diabetes (T1D), a chronic autoimmune disease, is characterized by the activation of autoreactive CD8^+^ T cells and autoantibodies that target and destroy insulin-producing β-cells in the pancreatic islets of Langerhans and insulin directly. A hallmark of T1D onset is insulitis, defined as the inflammation and eventual destruction of islet β-cells leading to a complete cessation of insulin production (6). The majority of T1D cases are diagnosed in children, with the greatest risk between the ages of five and seven and during puberty. Before diagnosis, elevated blood glucose (hyperglycemia) causes symptoms such as excessive thirst (polydipsia), excessive hunger (polyphagia), and excessive urination (polyuria). Upon diagnosis, T1D requires immediate and lifelong insulin treatments to prevent life-threatening acute and chronic complications (7).

The etiology of T1D remains poorly understood, with genetic, environmental, and immunological factors predicted to determine susceptibility and trigger disease onset. One established genetic risk factor is the presence of specific human leukocyte antigen (HLA) alleles, particularly HLA-DQB1*03:02, which facilitates the presentation of antigenic peptides that activate autoreactive T cells (8). It has been argued that T1D risk is increasing more rapidly than can be attributed to genetics alone. For instance, Jerram et al. (9) found that identical twins have a high discordance rate for the disease when they are over 15 years old at diagnosis. It has also been shown that HLA haplotype sharing between siblings does not account for diabetes risk (9, 10).

Immunological differences exist between individuals recently diagnosed with T1D and patients with established disease, some details of which implicate MAIT cells. For instance, in recently diagnosed children, the frequency and number of blood-derived MAIT cells were threefold lower than in non-diabetic controls. In contrast, no significant differences in MAIT cell frequencies were observed in children with established T1D (6). One interpretation of this pattern is that circulatory MAIT cells may be recruited to the pancreas and contribute to inflammation in early T1D onset.

Another immunological difference between recent-onset and established T1D involves the concept of immune aging. T1D progression has been associated with accelerated immune aging, marked by an increased frequency of CXCR3^+^ and programmed cell death-1-positive (PD-1^+^) cells among naïve and memory T cell subtypes. This phenomenon may result from the chronic inflammation and hyperglycemic stress that compound as T1D progresses, particularly when blood glucose is poorly controlled (11). The role of MAIT cells in this process, if any, remains elusive.

Lastly, individuals with T1D may experience partial remission (PR) shortly after diagnosis. PR involves a temporary reversal of hyperglycemia with reduced dependence on insulin treatment that subsides as the disease progresses. This phase may be driven by TIGIT^+^CCR7^-^Tregs, which suppress CD8^+^ T cells. The proportion of these Tregs is low in newly diagnosed patients, peaks during the PR phase, and declines as PR ends. CD226^+^CCR7^-^CD8^+^ T cell subsets exhibit the opposite trend, suggesting a temporary interaction between these T cell subtypes during PR (12). It is not known whether MAIT cells have a role in this phenomenon. In fact, hypotheses regarding the influence of MAIT cells on the onset and progression of T1D are difficult to test in humans due to the invasive nature of obtaining pancreatic biopsies, although emerging non-invasive cell-specific imaging technologies may provide answers (13). Another important consideration in human research is to specify a common criterion for a “recent” T1D participant as opposed to an “established” T1D patient.

Another approach to facilitate studying T1D-induced inflammation in the pancreas is to expand the range of biomarkers for T1D pathogenesis, particularly in the period between the detection of autoantibodies and detectable disease and prognostic markers (14). While circulatory cell populations provide some insight, cell phenotypes are influenced by environmental and age-related factors and do not adequately reflect cellular activity in peripheral lymphoid organs such as lymph nodes or in pancreatic islets (11). Can novel immunological elements of pancreatic pathogenesis, such as MAIT cell involvement, provide diagnostic and/or prognostic biomarkers for human T1D?

## The gut microbiome

### MAIT cells and the gut microbiome

In healthy individuals, barrier sites of immunity such as the epidermis, gut, lungs, and reproductive tract host a diverse population of commensal microbes collectively referred to as the microbiota. The relationship between these microbes, the most studied of which are bacteria, and the host is mutualistic. In the human gut, commensal bacteria such as Firmicutes, Bacteroidetes, and Actinobacteria (15) produce metabolites (e.g., short-chain fatty acids), nutrients (e.g., vitamin K), and digest cellulose while the host provides favorable conditions for bacterial growth (16). Gut microbiota also contribute to immune maturation and homeostasis by competing with pathogenic species for nutrients and space, a phenomenon known as ‘colonization resistance,’ (17, 18) and by promoting barrier integrity (19). Notably, early-life microbial colonization has been implicated in homeostatic immune development and long-term health effects on the host (20). For instance, Caesarean section (C-section), as opposed to vaginal delivery, disrupts the typical colonization of the neonatal gut and consequently has been linked to respiratory disorders, asthma, and T1D (21). Thus, suitable microbial colonization contributes to healthy immune development, and disruptions to this process can have lasting implications for immune-mediated diseases.

A healthy gut microbiome appears essential for MAIT cell development. At birth, MAIT cells in the thymus and cord blood are low in number and express a naïve phenotype. After birth, when MAIT cells have been exposed to metabolites from commensal bacteria colonizing the gut, the MAIT cell population expands and matures in the periphery, adopting a memory phenotype (22). Of note, the gut-resident Bacteroidetes are a significant riboflavin metabolite producer and MAIT cell stimulator (23), contributing to MAIT cell maturation in the gut lamina propria. In addition to commensal microbes, MAIT cells require interaction with MR1-expressing B cells. MR1 is important for MAIT cell selection and expansion, and B-cell-deficient patients lack MAIT cells (24).

Following maturation, MAIT cells can migrate to and reside in various barrier tissues, including the gut, through interactions with chemokines, chemokine receptors and tissue adhesion molecules (5). In barrier tissues, MAIT cells are positioned to respond to microbial-derived antigens.

The importance of the gut microbiome to MAIT cell development has been deduced with animal models. Constantinides et al. (20) identified MAIT cell reliance on the gut microbiome in mice by determining that germ-free (GF) mice possessed significantly fewer MAIT cells in their thymus, spleen, skin, lung, liver, and gut than specific-pathogen-free (SPF) animals. MAIT cell development can be stimulated in GF mice by colonization with riboflavin metabolite-producing bacteria. Constantinides et al. (20) colonized neonatal GF mice with the Enterobacteriaceae species *Proteus mirabilis*, a common murine gut commensal capable of synthesizing riboflavin metabolites. The colonization promoted MAIT cell development in the GF recipients’ skin, but it was seemingly age-restricted. Colonization of GF mice later in life with the same microbe failed to induce MAIT cell development in barrier tissues. Colonization of neonatal GF mice with *Lactobacillus johnsonii*, a microbe that lacks riboflavin synthesis enzymes, failed to promote MAIT cell development. The authors concluded that MAIT cell development in the skin may rely on early-life exposure to gut microbes capable of synthesizing riboflavin metabolites, such as Enterobacteriaceae, which cannot be substituted by exposure later in life. However, this study was limited to the assessment of skin MAIT cells. The experiment could be expanded to investigate the impact of colonization on MAIT cell populations in other barrier tissues, such as the gut, which *P. mirabilis* colonizes. If MAIT cell development and maintenance depend on interactions with these riboflavin metabolite-producing Enterobacteriaceae species, it would be reasonable to predict a higher density of MAIT cells in the gut where these bacteria colonize in high proportions.

In humans, preterm birth has been linked to an increased risk of developing T1D (25) and in the past decade, many centers have given preterm infants probiotics on the premise that biasing their colonization will reduce the risk of several diseases. However, and possibly at odds with promoting MAIT cells, one common outcome in these infants is reduced abundance of Enterobacteriaceae (26); whether other bacteria with the riboflavin synthesis pathway may prevail is not clear and deserves attention.

### Type 1 diabetes and the gut microbiome

The global incidence of T1D among adolescents and young adults increased from 7.78 per 100,000 individuals in 1990 to 11.07 per 100,000 in 2019 (27), with an average annual growth rate of 3-4% over the past three decades (28). In 2019, the highest T1D incidence rates were reported in European countries such as Finland, Norway, and Spain, as well as in Canada. However, the most significant incidence increases between 1990 and 2019 occurred in countries with mid-range socio-demographic indices. Although T1D has historically been prevalent in highly economically developed nations, the burden is expanding to more resource-limited regions (27). This trend raises the question of whether the rising T1D incidence is linked to environmental changes, particularly those affecting the gut microbiome.

The gut microbiome can be influenced by a number of factors throughout an individual’s life, beginning as early as birth. Infants delivered vaginally host bacterial communities that resemble their mother’s vaginal microbiota (e.g., *Lactobacillus*, *Prevotella*), while infants delivered by C-section acquired bacteria similar to those on the skin, such as *Staphylococcus* and *Corynebacterium* (29). Recently, Reznik et al. (21) have shown that C-section, as opposed to vaginal delivery, disrupts the typical colonization of the neonatal gut and consequently has been linked to respiratory disorders, asthma, and T1D.

The first documented connection between the gut microbiome and T1D was in 1993 when non-obese diabetic (NOD) mice showed differential diabetes outcomes based on their housing conditions (30). NOD mice are widely used as a model for T1D because the mice spontaneously develop a T1D-like condition characterized by the immune-mediated destruction of pancreatic β-cells (31). Since the observation in NOD mice, gut dysbiosis has emerged as a focus of investigation in human T1D.

Yuan et al. (32) reported that individuals with T1D exhibit decreased gut microbial diversity, a lower Firmicutes/Bacteroidetes ratio, and a deficiency in short-chain fatty acid-producing bacteria compared to non-diabetic controls. Hemoglobin A1C (HbA1C), fasting blood glucose, and markers of systemic inflammation - such as total white blood cell and neutrophil counts - were inversely associated with short-chain fatty acid-producing species, suggesting a correlation between the gut microbiota unique to those with T1D and the disrupted glucose metabolism and inflammation associated with the disease. This study assayed only for systemic inflammation and did not test for pancreatic inflammation, a hallmark of T1D.

Studying events in the inflamed human pancreas has significant obstacles. Human pancreatic analysis by biopsy is challenging and seldom performed in T1D research. One investigative solution to this problem involves the use of magnetic resonance imaging (MRI) combined with an FDA-approved ferumoxytol nanoparticle (13). This method relies on the engulfment of the nanoparticle by tissue-resident or recruited macrophages in the inflamed pancreas, resulting in tissue changes detectable and quantifiable by MRI. The approach has been demonstrated in NOD mice and was ready for human clinical trials at the time of publication. If successfully implemented, this MRI-based approach could provide insights into differences between the pancreas of individuals with T1D without requiring invasive tissue collection procedures.

Antibiotic treatment is a leading cause of gut dysbiosis, and the rise of autoimmune and allergic diseases coincides with the advent of antibiotics and resulting decrease in infectious diseases. Using mice again, the incidence rate of diabetes in NOD mice is 90.6% in females and 64.3% in males, yet following oral treatment with vancomycin from conception (via treated breeding pairs) through adulthood, incidence rates rose to 97.8% in females and 91.4% in males, a significant increase for males. Males also developed diabetes significantly sooner following treatment, with the median age of onset decreasing from 29.4 weeks to 22.4 weeks. The authors concluded that, while their findings cannot confirm that early-life antibiotic use in children increases their risk of autoimmune disease, the outcome supports the hygiene hypothesis - that limited/reduced exposure to infections early in life may increase the risk of autoimmune and allergic conditions (33), including T1D.

Still, NOD mice may be a flawed model from which to draw these conclusions. While NOD mice share key disease traits with humans with T1D, therapeutic strategies that have effectively delayed or prevented diabetes in NOD mice have not been successful in human trials (34, 35), suggesting underlying differences between the two species. One such important difference is that diabetes incidence in NOD mice occurs with a female-to-male sex bias that is seldom observed in humans with T1D. Castration of male mice increased diabetes incidence and testosterone treatment in female mice has a protective effect, findings that have not been reproducible in GF mice, suggesting that certain commensal bacteria may contribute to male protection (31). This pattern aligns with the findings from Candon et al. (33) that vancomycin treatment and subsequent microbiome disruption suppressed the male protection that was observed pre-treatment, resulting in increased diabetes incidence rates in male NOD mice, while females remained highly susceptible regardless of treatment.

Interestingly, when contents from the cecum were transferred from mature male to immature female NOD mice, the recipient females were protected against pancreatic islet inflammation, autoantibody production, and diabetes development (31). This protection correlated with increased testosterone levels in the recipient females, leading the authors to hypothesize that gut microbiota in NOD mice regulate sex hormones that, in turn, influence diabetes susceptibility.

Despite these compelling observations from mouse experiments, T1D in humans has not been confirmed to have a sex bias, contrasting other autoimmune diseases such as rheumatoid arthritis and multiple sclerosis. The latter have a female-to-male bias that diminishes over time as testosterone levels decrease, while T1D remains largely unbiased, likely due to the typical pre-pubescent onset (31). Human T1D having a pre-pubescent onset sheds light on another difference between the NOD model and humans; the lifetime of NOD mice is compressed, with puberty in mice occurring around 6 weeks while diabetes onset occurs after 14 weeks (31), implicating factors other than hormonal changes in human T1D incidence.

The prevailing published research indicates that an abnormal gut microbiome, particularly in early life, may increase the risk of developing T1D, although it is unconfirmed whether this includes impacts of the dysbiosis specifically on MAIT cells. There may be a relationship in T1D patients between gut dysbiosis and disease indicators such as HbA1C and systemic inflammation that could involve MAIT cells. While the gut microbiota appears to regulate sex-specific diabetes protection in NOD mice, this phenomenon is not believed to extend to human T1D.

## The association with MAIT cells in the gut and pancreas

### MAIT cells in the gut

Mucosal sites host a wide variety of immune cells but are especially populated with innate and innate-like T cells, such as γδ T cells, invariant natural killer T cells, and MAIT cells. Through the secretion of enzymes, cytokines, and mediators, these cells are positioned to respond to infectious agents and induce tissue repair (36). The small and large intestines, both MAIT cell-enriched organs, host diverse microbiota that produce MAIT cell-activating ligands (20). At steady-state, MAIT cells in the gut play a role in maintaining mucosal integrity by producing IL-17 and IL-22, which support epithelial cell survival and proliferation, stimulate mucus production by goblet cells, and regulate occludin expression, a tight junction protein critical to barrier permeability (36).

At steady-state in the gut, MAIT cells exhibit low cytotoxicity but can promote tissue repair through TCR-dependent activation and production of IL-17 and IL-22 (36). TCR-activated MAIT cells demonstrate increased expression of tissue-repair genes and promote the growth of a model human intestinal epithelial cell line (37). These functions, occurring at steady-state, are associated with a diverse, healthy microbiome.

At steady-state, the availability of certain substrates in the gut, particularly oxygen, is regulated to maintain the composition of the gut microbiome. This regulation is achieved in part by the colonic epithelium, which consumes much of the oxygen delivered by the blood, creating an environment in the colonic lumen that favors the growth of anaerobic Firmicutes and Bacteroidetes species. When oxygen is in excess, dysanaerobiosis occurs, a form of microbiome dysbiosis that favors the proliferation of facultative anaerobes like Enterobacteriaceae (37). Dysanaerobiosis is associated with gut pathologies such as inflammatory bowel disease (IBD) and colorectal cancer, likely due to the ulcers and gastrointestinal bleeding that occur as a result of prolonged colonic inflammation, feeding oxygenated blood directly into the colonic lumen (37, 38). Riboflavin is a precursor to co-enzymes required for the aerobic electron transport chain (39), meaning an aerobic environment may increase the local riboflavin requirement. This requirement is shouldered by the gut microbiota, leading to an increase in metabolites available for MAIT cell activation (37). Consistent with this, MAIT cells are activated in various gut pathologies associated with dysanaerobiosis including ulcerative colitis (40) and colorectal cancer (41), and the findings from Constantinides et al. (20) suggest that a gut microbiome rich in Enterobacteriaceae, the facultative anaerobe, may contribute to MAIT cell development and maintenance.

In a pathological state, such as inflammation or infection, MAIT cell frequency increases in the affected tissue, such as the gut, due to increased migration to the site through the blood and/or local proliferation. Under these conditions, MAIT cells secrete proinflammatory cytokines TNF, IL-17, and IFN-γ, exerting anti-pathogen activity, inhibiting viral replication, and preventing the proliferation of cancerous cells. This activation is also associated with an up-regulation of cytotoxic effectors such as granzyme B, perforin, and granulysin (36). Wu et al. (5) review instances of MAIT cell recruitment to inflamed mucosal sites. In one example, MAIT cell frequency in patients with IBD was lower in the blood but higher in the inflamed colon than in healthy donors (42). Murine models have also provided insight into potential roles for MAIT cells in intestinal pathology. Mice lacking MAIT cells are more susceptible to chronic colitis and inflammation-induced colorectal cancer, and MAIT cells obtained from models of chronic intestinal inflammation clustered due to common expression of the anti-inflammatory cytokine transforming growth factor-beta 1 (TGF-β1) and receptor cytotoxic T-lymphocyte-associated protein 4 (CTLA-4). This potentially protective role for MAIT cells in pathological contexts in the gut is also supported by the finding that, in a mouse model of colitis, activated MAIT cells expressed tissue repair genes and secreted barrier-promoting factors, such as hypoxia-inducible factor 1 subunit alpha (HIF1A) (37). Together, these findings suggest that MAIT cells under steady-state, and potentially in dysanaerobiosis-associated pathologies such as an inflamed colon, play a protective role in the gut through tissue repair and protection against further inflammation. However, it is unclear whether MAIT cells associated with T1D-related inflammation exhibit the same protective role.

### MAIT cells in the pancreas

Not surprisingly, MAIT cell function in the healthy human pancreas is poorly understood as most pancreatic studies are concerned with pathological states. Here again, rodent models lead, with the NOD mouse model providing insights into the relationship between MAIT cells and the pancreas in T1D. Rouxel et al. (6) conducted an experiment classifying diabetes progression in NOD mice into three stages based on age: early (6–7 weeks old), pre-diabetic (15–17 weeks old), and diabetes onset (17 weeks and onward). As the disease progressed, the frequency of MAIT cells in the pancreatic islets increased. To assess whether these recruited MAIT cells contributed to diabetes progression, the authors analyzed cytokine production by the cells in the pancreatic islets across the three stages of disease development. The results showed stage-specific differences in cytokine production, with diabetic NOD mice exhibiting upregulated IFN-γ and granzyme B, known to induce pancreatic β-cell death (43). These findings suggest that MAIT cells recruited to the pancreas during diabetes progression may exacerbate β-cell destruction (6).

## The relationship between type 1 diabetes progression and MAIT cell populations

Associations between the gut microbiome and T1D, as well as between the gut microbiome and MAIT cells, have been observed. Therefore, a relationship is predicted to exist between T1D and MAIT cell populations in the blood (where complications such as hyperglycemia and elevated HbA1C manifest), the pancreas, which is the primary immunopathological site in T1D, and the gut, a critical barrier tissue protective against bacterial and viral pathogens and the host of a rich, diverse microbiome.

### MAIT cells and type 1 diabetes – the blood and the pancreas

MAIT cell population distinctions have been identified between children with recently diagnosed T1D (i.e., within 10 days of their first insulin injection) and those with established disease. Rouxel et al. (6) reported that the frequency and number of MAIT cells in the peripheral blood of children with recent-onset T1D were threefold lower than in non-diabetic controls, bringing the frequency of MAIT cells among CD3^+^ T cells in the blood to less than 1%. In contrast, no differences were observed between children with established T1D and controls.

Phenotypic differences in the MAIT cells of children with T1D were also observed. Children with recent-onset T1D expressed less chemokine receptor 6 (CCR6), a T cell homing receptor, and less B-cell lymphoma-2 (BCL-2), an anti-apoptotic molecule, than control children, suggesting the reduction in circulating MAIT cells early in T1D onset could result from directed recruitment to inflamed tissues (the pancreas)?, or death by apoptosis (6). If migration is the primary cause behind the reduction, it raises questions of whether T1D-mediated MAIT cell activity in the pancreas is transient or chronic, and from which site the pancreatic MAIT cells originate. Are these cells predominantly circulatory and recruited to the pancreas, or is the blood the channel for migration from another mucosal tissue, such as the gut?

When analyzed for cytokine and granzyme B production, blood MAIT cells from recently diagnosed children produced less IFN-γ but higher levels of TNF, IL-4, and the protease granzyme B compared to non-diabetic controls. In children with established T1D, MAIT cells produced these mediators at moderate levels (6). Interestingly, the authors observed that the frequency of granzyme B-producing MAIT cells decreased as the age of diagnosis increased in the recent-onset cohort – children diagnosed at a younger age had more granzyme B-producing MAIT cells upon diagnosis. This suggests the disease may be more cytotoxic and aggressive in younger children. Additionally, the frequency of the granzyme B-producing MAIT cells inversely correlated with HbA1C results, but this was only observed at disease onset and not during later stages of disease progression. The inverse correlation is logical; if granzyme B-producing MAIT cells contribute to a more aggressive disease state at T1D onset, diagnosis may occur earlier (6). This would allow insulin treatment to begin sooner, lowering blood glucose and HbA1C levels. However, the authors did not specify whether HbA1C measurements were taken at diagnosis or after a few months of insulin treatment.

Finally, to investigate peripheral MAIT cell cytotoxic potential against pancreatic β-cells, Rouxel et al. (6) analyzed how cytokines associated with inflamed pancreatic islets during T1D progression (such as IL-1β, IFN-γ, and TNF) affected MR1 expression. They found the cytokines upregulated MR1 expression on a model cell line for β-cells, EndoC-βH1. Considering that MAIT cells can be activated through TCR interactions with ligands presented on MR1, the authors co-cultured MAIT cells with EndoC-βH1 and found that MAIT cells induced MR1-dependent apoptosis in the β-cell line, pointing to a potential mechanism of β-cell destruction by MAIT cells. However, the MAIT cells used in this experiment were purified from non-diabetic donors, which may limit the applicability of the findings to T1D patients. Yet overall, these findings suggest that in children recently diagnosed with T1D, peripheral MAIT cells may be recruited to the inflamed pancreas, contribute to β-cell death, and worsen disease progression (6).

### MAIT cells and adult T1D

The findings from Rouxel et al. (6) suggest that granzyme B-producing MAIT cells exerting cytotoxic functions are inversely associated with age at diagnosis. Younger children exhibited the highest frequencies of these cells, aligning with studies showing that pancreatic β-cell destruction occurs more rapidly in children and that diabetic ketoacidosis is more prevalent in juvenile-onset T1D compared to adult-onset (44).

To further investigate the link between age, blood and pancreatic MAIT cell populations, and T1D, a study analyzing the frequency, phenotype, and function of MAIT cells in the blood of adults with recent-onset (2–14 days post-diagnosis) or established (2+ years post-diagnosis) T1D was reviewed (44). Among CD3^+^ cells, the proportion of peripheral MAIT cells was not significantly different between non-diabetic controls, adults with recent-onset T1D, and adults with established T1D. Unlike in children, the frequency of MAIT cells expressing homing molecules such as CCR6 was comparable across all three groups, suggesting no differential migration based on disease progression. However, the frequency of CD25^+^ MAIT cells, an activation marker, was increased in recently diagnosed adults compared to controls, suggesting an activated MAIT phenotype. Interestingly, unlike in children, the frequency of granzyme B-producing peripheral MAIT cells positively correlated with HbA1C levels in recently diagnosed adults (44). If T1D is less aggressive in adults, as noted by Karjalainen et al. (45), adults may experience delayed diagnoses with HbA1C levels that progressively rise (44). In contrast, adults with long-term T1D exhibit MAIT cells with elevated expression of CD25 and PD-1, an exhaustion marker, compared to controls. The authors also observed that MAIT cells from adults with long-term T1D produced lower amounts of Th1 proinflammatory cytokines (IL-2, IFN-γ, TNF). Therefore, while MAIT cells persist in the blood of individuals with both recent-onset or established T1D, MAIT cells exclusively associated with long-term disease exhibit signs of exhaustion. In other words, the phenotype and functions of peripheral MAIT cells in adults with T1D evolve with disease progression (44).

The exhaustion exhibited by MAIT cells in adults with long-term T1D is a result of chronic activation, potentially in response to the inflammation associated with poor blood glucose management (44). This hypothesis supports the concept of immune aging as a consequence of T1D progression (11). However, all participants in this study had well-controlled blood glucose levels and no T1D-related complications (44). Future research focusing on PD-1^+^ peripheral MAIT cell populations in individuals with long-term T1D and poorly managed blood glucose levels could provide further insights into the mechanisms that drive immune aging in T1D.

## MAIT cells and type 1 diabetes – the gut connection

**Figure 1:**
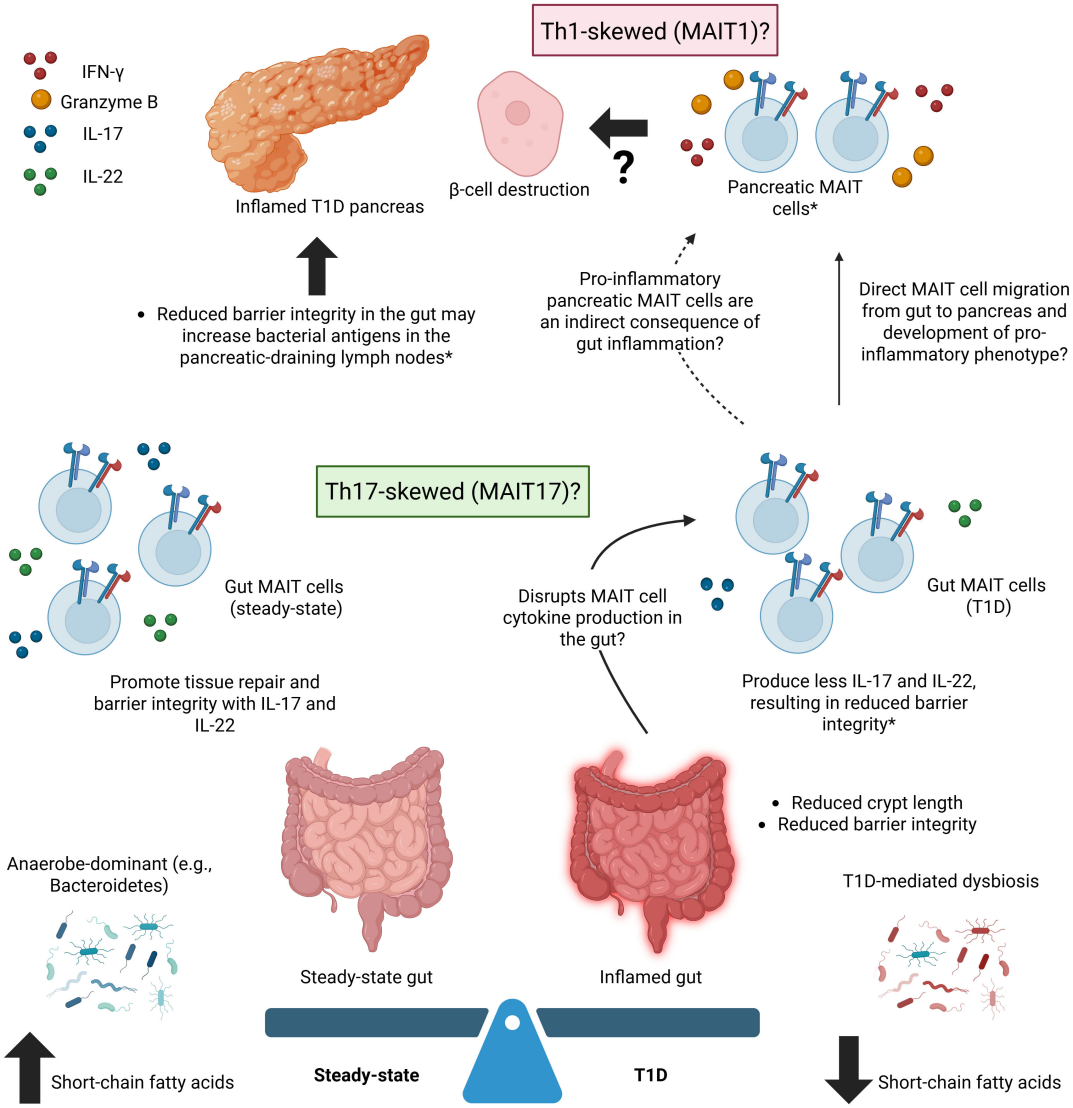
A Gut-Pancreas Connection in Type 1 Diabetes – Potential Roles for MAIT Cells. Potential roles for MAIT cells in connecting the inflamed, dysbiotic gut to the inflamed pancreas in type 1 diabetes (T1D). T1D is associated with an increased frequency of MAIT cells producing inflammatory factors (e.g., IFN-γ, granzyme B) in the pancreas, an inflamed gut, and microbiome dysbiosis. Gut MAIT cells are typically protective, regardless of inflammation, while pancreatic MAIT cells are generally only associated with pancreatic pathology. MAIT cells in the T1D pancreas may be a result of the direct migration of protective MAIT cells from the gut to the pancreas that adopt a pro-inflammatory phenotype or of gut inflammation and dysbiosis that indirectly lead to the recruitment of pro-inflammatory MAIT cells to the pancreas from a source that remains unknown. *refers to findings in mouse models. Created in BioRender. Fraser, D. (2025) https://BioRender.com/bjd8oau.

The pancreas is not the only organ implicated in T1D – the gastrointestinal tract, or gut, is altered in both human T1D patients and lab animal models of diabetes. The alterations primarily affect barrier integrity, with T1D being associated with reduced crypt length, decreased expression of proteins that secure tight junctions, and chronic gut inflammation (46). Yet, despite indirect or incidental evidence, the relationship between the gut and pancreas in T1D remains elusive. There may be a connection between gut dysbiosis and the disease indicators and systemic inflammation associated with T1D, but the capacity in which MAIT cells are involved is unclear. It is possible that the protective MAIT cells activated in the inflamed gut migrate to the pancreas and adopt a pro-inflammatory phenotype, potentially contributing to b-cell death, or that a dysbiotic, inflamed gut can activate pro-inflammatory MAIT cell activity in the pancreas through indirect effects (**Figure 1**). *In vivo* MAIT cell tracking in the NOD mouse model, potentially incorporating Kaede photoconvertible protein expression (47), throughout diabetes onset and progression could shed light this relationship.

While certain phenotypic and functional characteristics of MAIT cells imply they play a protective role in maintaining gut integrity and regulating the autoimmune response associated with T1D, this protection is thought to diminish as the disease progresses. Rouxel et al. (6) demonstrated that factors mediating gut integrity weaken or are lost with diabetes progression in NOD mice. MAIT cells in the pancreatic islets of diabetic NOD mice produced more granzyme B than pre-diabetic mice and IFN-γ, both of which have been implicated in β-cell death (43). MAIT cells in the ileum of diabetic NOD mice produced less IL-17 and IL-22 (6), cytokines that are typically associated with maintenance of intestinal homeostasis.

Of note, the presence of IL-17 and IL-22 has implications for other immune cells, particularly Th17 and γδ T cells. In the presence of IL-17, naïve CD4+ T cells can differentiate into Th17 cells. These cells also play an important and potentially pathological role in T1D, indirectly implicating MAIT cells. Treatment with antibodies against IL-17A has been shown to inhibit disease in NOD mice (48), and Th17 cells have been shown to drive pancreatic inflammation and their transfer into NOD.scid mice induced diabetes, but only after converting to Th1 cells (49). IL-17 is also secreted by γδ T cells at high levels, promoting inflammation and amplifying the local immune response (50). IL-22, which, along with IL-17, is produced by Th17 cells and supports homeostasis, helps remodel epithelial tissue, and supports barrier integrity (51).

In an adaptation of the NOD model that would result in MAIT cell-deficient mice, Rouxel et al. (6) engineered *Mr1^-/-^* NOD mice. The mice showed significantly reduced occludin expression, an abnormally high amount of mucus in goblet cells, and greater gut permeability than *Mr1^+/-^* counterparts. These alterations in gut integrity led to a higher presence of bacterial 16S DNA in the pancreatic-draining lymph nodes, suggesting a ‘leaky’ gut that may exacerbate T1D onset and development by activating dendritic cells and promoting autoreactive, cytotoxic T-cell responses in the pancreatic islets. These findings suggest that MAIT cells may support a protective role in the gastrointestinal tract under normal conditions, which, if disrupted, may contribute to T1D progression and a pathological role for MAIT cells in the pancreas, contributing to the interplay between the gut, T1D, and inflammation (6).

MAIT cells adopting a protective role in the gut and a pathological role in the pancreas could arise from various mechanisms, including exposure to cytokines, antigens, or interactions with other immune cells. In support of a protective role in the gut, as previously described, MAIT cells are activated by riboflavin metabolites presented by MR1 and express a sufficient level of proinflammatory cytokines and cytotoxic factors to protect mucosal barriers. These MAIT cells are primarily of MAIT17 (RORγt^+^) lineage, while the IFN-γ-secreting MAIT1 (T-bet) cells typically localize to the liver and spleen. The differentiation between lineages may, in part, be regulated by microRNA-155, an epigenetic mechanism (52). Overall, the production of IL-17 and IL-22 in the gut helps sustain epithelial integrity and promote wound repair (37). However, if this protective effect is compromised, potentially from gut microbiome dysbiosis or pancreatic inflammation associated with T1D, the resulting gut MAIT cell populations and their IL-17 and IL-22 production may be affected and T1D onset/progression may be exacerbated.

Evidence observed regarding human peripheral and murine peripheral and pancreatic MAIT cells aligns with potential dysregulation of MAIT IL-17 and IL-22 production in T1D patients. In NOD mice, MAIT cells became increasingly localized to the pancreas as diabetes progressed and were associated with an increase in IFN-γ and granzyme B (6). As IFN-γ is more characteristic of a MAIT1 response, is it possible that the pancreatic MAIT cells shifted from a MAIT17 to MAIT1 phenotype in the NOD mice, leading to reduced IL-17 production? A Th1-skewed environment in the pancreas, characterized by IL-1β, IFN-γ, and TNF, in human children upregulates MR1 expression by a model line for pancreatic β cells, which in return is destroyed by MAIT cells (6). IL-17 and IL-22 production may also be influenced by the abundance of other immune cells, such as Th17 and γδ T cells, or by cytokines IL-12 and IL-18, which have been shown to induce the production of IL-17A (53). However, our understanding of MAIT phenotypes and their associated cytokines and mediators is limited by a lack of technology suitable for direct examination of humans.

Regarding the gut-pancreas axis and potential MAIT cell interactions between the two sites, it will be important to examine the gut permeability in human participants with recent-onset and long-term T1D. Methods such as sugar absorption tests are available and widely used in gastrointestinal research. In this method, gut permeability is measured by urinary excretion of orally-consumed sugar probes, such as lactulose/mannitol (LM). Briefly, a small (mannitol) and a large (lactulose) molecule are ingested and the ratio of levels recovered in the urine is a measure of permeability along the intestines. Limitations include protocol variability, interfering factors such as hydration status and renal function, and complexities with interpretation (54, 55). One concern for T1D cohorts is that a sugar-based test may pose a risk for blood sugar spikes, but interestingly, there is a precedent for applying the test on T1D patients without any reported blood sugar issues (56). The authors found that individuals with pre-clinical, recent-onset, or long-term T1D showed increased intestinal permeability to lactulose, suggesting damage to the small intestinal barrier even before clinical onset of the disease (56).

Alternative, non-sugar-based tests exist - biomarkers such as serum zonulin have also been used to assess gut integrity. Zonulin is a protein that interacts with tight junctions between epithelial cells in the intestinal lining and has been proposed as a biomarker for intestinal permeability (54). Serum zonulin levels are currently determined using commercial enzyme-linked immunosorbent assays (ELISA), but it has been observed that these assays do not accurately measure zonulin concentration and detect unrelated proteins. There is also limited evidence that serum zonulin levels, as measured currently, correlate with findings from more established tests such as LM (57). Of note, individuals with T1D at pre-clinical and clinical stages have shown increased concentrations of serum zonulin that correlated with increased intestinal permeability compared to age-matched controls (58). These findings align with those observed with the LM test (56), supporting a potential connection between increased intestinal permeability and T1D, though further development of the respective tests is advised before drawing conclusions. Investigating potential changes in gut permeability as T1D progresses could provide insight into gut-related pathologies and their effect on T1D progression, assess one's risk for developing gut-related conditions as a T1D patient, and perhaps even identifying gut permeability as an indirect approach to mitigating the inflammation of T1D.

Lastly, there is limited research on the connection between MAIT cells and the partial remission phase often observed shortly after T1D onset. While most evidence suggests that MAIT cells have pathological effects in the pancreas, their protective role in the gut raises the possibility that the cells may exhibit temporary protective functions in the pancreas during early disease stages. Investigating this could provide insights into the short-term reversal of T1D symptoms in newly-diagnosed individuals.

## The feasibility of a MAIT cell-based biomarker for type 1 diabetes

It is evident that MAIT cell alterations exist in individuals with T1D at disease onset and throughout disease progression. Biomarkers that focus on circulating non-MAIT immune cells exist for T1D autoimmunity, but do not necessarily reflect the conditions in the pancreas and are influenced by age- and environment-related factors (11). T-cell biomarkers are established in T1D research. For instance, islet-reactive CD8+ T-cell frequency is increased in the pancreas of T1D patients compared to controls, but this marker requires further clinical validation (59, 60). The isolation and detection of T cells can also be challenging – autoreactive T cells frequently migrate from the blood to secondary lymphoid organs and insulitic lesions, impacting blood levels (59). A model that can detect abnormal MAIT cells in the blood – specifically phenotypes associated with T1D onset - could be a valuable complement or alternative. Such a model could also help determine whether these altered MAIT cell phenotypes and functions are a consequence of T1D onset and progression, or whether they precede T1D pathology and serve as indicators of an individual’s risk of developing the disease. Accordingly, phenotypic markers of MAIT cells have been considered as potential biomarkers for T1D. The expression of various surface and intracellular molecules distinguished MAIT cells between children recently diagnosed with T1D, children with long-term T1D, and children without T1D. Four surface markers in particular - CCR6, CD25, PD-1, and CD56 - were combined into a predictive model (6). The authors subsequently characterized MAIT cells in adults at risk of developing T1D (i.e., direct relatives of T1D patients with at least two positive autoantibodies). These individuals displayed increased frequencies of CD25^+^ and PD-1^+^ MAIT cells and a lower frequency of CCR6^+^ MAIT cells compared to low-risk adults, though the study was limited by its small sample size (*n* = 11) (6). It is also unclear whether the adult participants, excluding a heightened risk for T1D, possessed any microbial dysregulation (e.g., microbiome dysbiosis, infection) that could confound these results. Nor were their ages and sex reported, leaving doubt over whether their pre-disposition to T1D was the sole reason for their unique MAIT cell phenotypes.

Models are most useful if they predict disease onset before the development of classic T1D symptoms. The fact that T1D has no cure means high-risk individuals could be made aware of their impending diagnosis with no current means to alter their fate, in which case it can be argued that a predictive model may cause more psychological turmoil than comfort. On the other hand, as we learn more about MAIT cell participation in the disease, MAIT biomarkers could be the target of intercepting disease progression. One example may be to reduce the cases of diabetic ketoacidosis that often precede diagnosis, especially in those who experience minimal hyperglycemia symptoms.

## Conclusions

MAIT cells exist at the intersection of T1D, pancreatic pathology, inflammation, and the gastrointestinal mucosa (**Figure 1**), demonstrating protective and pathogenic roles across various organs and disease states. It is evident that MAIT cells play a critical role in maintaining intestinal barrier integrity through their production of IL-17 and IL-22, and disruption of this function is linked to autoimmune diseases, including T1D. The exact mechanism by which T1D is connected to this process, and whether the role of T1D is causative or consequential, is still unclear. MAIT cell changes may be driven by gut microbiome dysbiosis in individuals with T1D, or by pancreatic inflammation arising from the autoreactive T cell destruction of insulin-producing β-cells. These factors may shift MAIT cells away from providing a protective role in the gut, instead contributing to chronic inflammation and increased permeability (observed predominantly in murine models).

Phenotypic changes have been observed in MAIT cells in patients with T1D, and these changes likely occur at MAIT cell maturity rather than during differentiation. MAIT cells mature in the thymus, where TCR rearrangements and assembly occur. Upon thymic egress, MAIT cells enter the circulation, migrate to the periphery, and adopt diverse functions based on exposure to different contextual signals. Early during tissue damage, MAIT cells are recruited to an inflammatory site where the cells proliferate and are activated by mucosal microorganisms or inflammatory mediators, leading to the secretion of tissue repair factors such as IL-17 and IL-22. This is a likely explanation for MAIT cells’ protective role in the gut, which may be intercepted by the pancreatic inflammation or microbiome dysbiosis associated with T1D. Changes in MAIT cell frequency and phenotype, influenced by age and disease progression, have been observed in peripheral blood, suggesting activation and recruitment to the pancreas during T1D onset. However, whether MAIT cell infiltration into the pancreas in T1D is transient, with peripheral MAIT cell numbers recovering due to proliferation, or whether these cells leave the pancreas and return to circulation as T1D progresses, remains to be determined. For now, murine models lead research related to the interplay between MAIT cells, the gut, T1D, and inflammation, but human longitudinal studies tracking MAIT cell proliferation and localization that implement recent technologies like pancreatic imaging could begin to address this question.
